# Radon-220 diffusion from ^224^Ra-labeled calcium carbonate microparticles: Some implications for radiotherapeutic use

**DOI:** 10.1371/journal.pone.0248133

**Published:** 2021-03-04

**Authors:** Elisa Napoli, Tina B. Bønsdorff, Ida Sofie Jorstad, Øyvind S. Bruland, Roy H. Larsen, Sara Westrøm

**Affiliations:** 1 Oncoinvent AS, Oslo, Norway; 2 Institute of Clinical Medicine, University of Oslo, Oslo, Norway; 3 Department of Radiation Biology, Institute for Cancer Research, Oslo University Hospital, Oslo, Norway; 4 Department of Oncology, Oslo University Hospital, Oslo, Norway; TRIUMF, CANADA

## Abstract

Alpha-particle emitting radionuclides continue to be the subject of medical research because of their high energy and short range of action that facilitate effective cancer therapies. Radium-224 (^224^Ra) is one such candidate that has been considered for use in combating micrometastatic disease. In our prior studies, a suspension of ^224^Ra-labeled calcium carbonate (CaCO_3_) microparticles was designed as a local therapy for disseminated cancers in the peritoneal cavity. The progenies of ^224^Ra, of which radon-220 (^220^Rn) is the first, together contribute three of the four alpha particles in the decay chain. The proximity of the progenies to the delivery site at the time of decay of the ^224^Ra-CaCO_3_ microparticles can impact its therapeutic efficacy. In this study, we show that the diffusion of ^220^Rn was reduced in labeled CaCO_3_ suspensions as compared with cationic ^224^Ra solutions, both in air and liquid volumes. Furthermore, free-floating lead-212 (^212^Pb), which is generated from released ^220^Rn, had the potential to be re-adsorbed onto CaCO_3_ microparticles. Under conditions mimicking an *in vivo* environment, more than 70% of the ^212^Pb was adsorbed onto the CaCO_3_ at microparticle concentrations above 1 mg/mL. Further, the diffusion of ^220^Rn seemed to occur whether the microparticles were labeled by the surface adsorption of ^224^Ra or if the ^224^Ra was incorporated into the bulk of the microparticles. The therapeutic benefit of differently labeled ^224^Ra-CaCO_3_ microparticles after intraperitoneal administration was similar when examined in mice bearing intraperitoneal ovarian cancer xenografts. In conclusion, both the release of ^220^Rn and re-adsorption of ^212^Pb are features that have implications for the radiotherapeutic use of ^224^Ra-labeled CaCO_3_ microparticles. The release of ^220^Rn through diffusion may extend the effective range of alpha-particle dose deposition, and the re-adsorption of the longer lived ^212^Pb onto the CaCO_3_ microparticles may enhance the retention of this nuclide in the peritoneal cavity.

## Introduction

Cancer therapy with radionuclides has been the recipient of increased interest, and several beta- and alpha-particle emitter-based therapeutic radiopharmaceuticals have either been approved or are undergoing clinical investigation [1–7]. The radionuclides that are used include the beta emitters ^89^Sr, ^90^Y, ^131^I, ^153^Sm, ^177^Lu and beta-emitting ^212^Pb, which generates alpha-emitting progenies, as well as the alpha emitters ^211^At, ^213^Bi, ^223^Ra, ^225^Ac, ^224^Ra and ^227^Th. In general, long-range, low linear energy transfer (LET) beta emitters are believed to be more suitable for the treatment of larger tumors than short-range, high-LET alpha emitters, which are considered to be more effective for the treatment of micrometastases and single-cell diseases [8].

**Fig 1:**
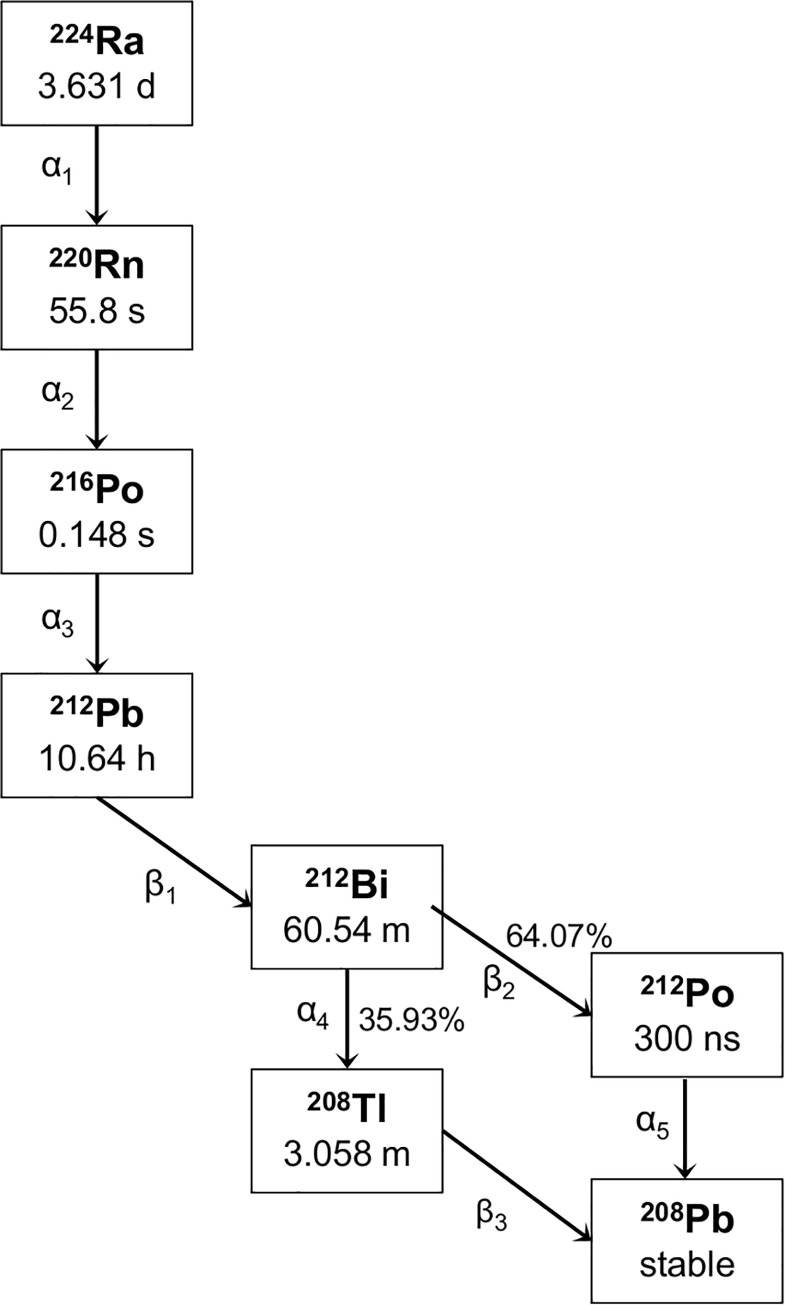
Decay chain of ^224^Ra and progenies to stable ^208^Pb. Half-life data are taken from the Decay Data Evaluation Project [10].

From a logistical point of view, ^224^Ra has a convenient half-life of 3.63 days [9, 10]. It decays via several radioactive progenies, producing four alpha particles and two beta particles (Table 1 and Fig 1). Recently, it has been subject of preclinical [11–15] and clinical [16–18] research for its potential use in antitumor agents. While the properties related to high-LET radiobiology [19] make ^224^Ra a potent cytotoxic agent, there are some concerns regarding the fate of its progenies *in vivo* as daughter nuclides can distribute differently than a parent because of differing biological affinities. For the brachytherapy application called diffusing alpha-emitters radiation therapy (DaRT) in which ^224^Ra-loaded wires are implanted into solid tumors, the distribution of progenies both within the tumor and in normal tissues have been examined [13, 20]. The release of progenies from one such ^224^Ra source has been shown to have a therapeutic effect in a region of 5–7 mm in diameter.

**Table 1:** Details of the nuclear decay data for ^224^Ra and its daughters indicating x- and γ-lines with 1% or higher abundance and divided into two columns: one for energies in the 60–110 keV detection window and the second for energies above 110 keV.

Nuclide	Half life	Daughter nuclide	x- and γ-lines, keV (Abundance)			
			60–110 keV		> 110 keV	
^**224**^**Ra**	3.631 days	(Rn)	None		241.0	(4.12)%
^**220**^**Rn**	55.8 s	(Po)	None		None	
^**216**^**Po**	0.148 s	(Pb)	None		None	
^**212**^**Pb**	10.64 h	(Bi)	74.8	(10.1%)	238.6	(43.6%)
			77.1	(16.9%)	300.1	(3.18%)
			86.8}	(5.77%)		
			87.3}			
			89.8}			
			89.7}	(1.77%)		
			90.1}			
			90.4}			
^**212**^**Bi**	60.54 min	(Po)/(Tl)	None		727.3	(6.65%)
					785.4	(1.11%)
					1620.7	(1.51%)
^**212**^**Po**	300 ns	(Pb)	None		None	
^**208**^**Tl**	3.058 min	(Pb)	72.8	(2.03%)	277.4	(6.6%)
			75.0	(3.42%)	510.7	(22.5%)
			84.5}	(1.17%)	583.2	(85.0%)
			84.9}			
			85.5}			
					763.5	(1.80%)
					860.5	(12.4%)
					2614.5	(99.8%)

All data were taken from the Decay Data Evaluation Project [10]. To compare the x-ray and gamma incidences between the radionuclides at equilibrium in the decay series, the branching factor (see Fig 1) for ^212^Bi, ^212^Po and ^208^Tl must be considered.

We have previously described the use of a suspension of calcium carbonate (CaCO_3_) microparticles as carriers for ^224^Ra and its progenies [21]. This novel application is designed to treat disseminated micrometastatic cancers, such as peritoneal carcinomatosis following intraperitoneal (IP) administration. Radium-224 adsorbed on CaCO_3_ microparticles has demonstrated antitumor activity against ovarian cancer xenografts in the peritoneal cavity of mice [11, 15]. Because of the multiple alpha-emitting daughters of ^224^Ra, it is important to investigate the interaction of these progenies with the carrier compound. For example, ^212^Pb, the progeny of ^224^Ra with the longest half-life in the decay chain (10.64 h [9, 10], Fig 1), may reach systemic circulation if it is prematurely released from the CaCO_3_ microparticles. A release of ^212^Pb from the carrier compound can influence the dose delivered to the target area and hence reduce the therapeutic effect of the product. Therefore, the behavior of the noble gas ^220^Rn, the immediate daughter of ^224^Ra and the grandparent of ^212^Pb in the decay chain, is of particular interest. Because it is gaseous, ^220^Rn may diffuse away from the CaCO_3_ microparticles and mediate a re-localization of the radioactivity.

In this study, we explored some fundamental product properties related to the two critical progenies, ^220^Rn and ^212^Pb, when CaCO_3_ microparticles are used as a carrier compound for ^224^Ra. The diffusion of ^220^Rn from the microparticles was investigated in both air and liquid phases. The fate of ^212^Pb subsequent to its release due to the diffusion of ^220^Rn was also studied under conditions mimicking an *in vivo* environment. Further, CaCO_3_ microparticles labeled with ^224^Ra through either surface adsorption or inclusion into the bulk of the microparticles were hypothesized to impact ^220^Rn diffusion and thus evaluated for their therapeutic effect in mice following the IP inoculation of the human ovarian cancer cell line ES-2.

## Materials and methods

### Extraction of ^224^Ra

Radium-224 was extracted via a ^228^Th source from Eckert and Ziegler (Braunschweig, Germany) or Oak Ridge National Laboratory (Oak Ridge, TN, USA) through previously published methods [21, 22]. In brief, the ^228^Th was immobilized on a column containing DIPEX^®^ (Eichrom Technologies LLC, Lisle, IL, USA) actinide resin. After allowing time for ingrowth, the ^224^Ra was eluted in 1 M HCl and evaporated to dryness. For subsequent use in radiolabeling, the residue was dissolved in 0.1 M HCl and pH adjusted to between 5 and 6 through the addition of NH_4_OAc (Merck, Darmstadt, Germany) to a final concentration of 0.5 M. The ^224^Ra was always at or close to equilibrium with progenies when used for labeling of the CaCO_3_ microparticles.

### Preparation of ^224^Ra-labeled CaCO_3_ microparticles

The ^224^Ra-labeled CaCO_3_ microparticles were prepared by two different procedures: (1) the adsorption of ^224^Ra onto the surfaces of pre-manufactured CaCO_3_ microparticles and (2) the incorporation of ^224^Ra into the bulk during CaCO_3_ microparticle production.

The CaCO_3_ microparticles that were subsequently used for surface labeling with ^224^Ra were prepared by a spontaneous precipitation process. In short, equal volumes of 0.33 M CaCl_2_ (Merck) and 0.33 M Na_2_CO_3_ (Merck or VWR International, Radnor, PA, USA) were mixed either by magnetic or overhead stirring. The microparticles were collected by centrifugation, subsequently dried in an oven for 1 h at 180°C and stored as a dried powder. In addition, a batch of CaCO_3_ microparticles was purchased from PlasmaChem GmbH (Berlin, Germany). In some experiments, the additive polyacrylic acid (PAA, average M_w_ ~250 000, 35% wt. in H_2_O, Sigma-Aldrich) was used to coat the CaCO_3_ microparticle surface at a ratio of 1.3 μL PAA solution per microparticle mg and added towards the end of the microparticle crystallization process. All types of microparticles had a mainly spherical geometry with volume-based median diameters ranging from 3–7 μm when representative batches were measured by laser diffraction (Mastersizer 3000, Malvern Instruments Ltd, Worcestershire, UK). Two microparticle batches, produced with and without PAA coating respectively, were also analyzed for visualization of crystal shape and surface morphology with scanning electron microscopy (SEM) performed at Particle Analytical (Hørsholm, Denmark) with a Leica Stereoscan 360. The results are presented in S1 Table.

For the surface radiolabeling, the microparticles were washed three times with water and two times with 0.1 M Na_2_SO_4_ (Merck) before dispersion in either 0.9% NaCl or a sucrose solution (composed of 94 mg/mL sucrose from Sigma-Aldrich, St. Louis, MO, USA and 2 mg/mL Na_2_SO_4_) as previously described [21]. Subsequently, ^224^Ra solution was added along with 0.004–0.3 w/w% Ba^2+^ and 0.3–0.6 w/w% SO_4_^2−^ relative to the amount of CaCO_3_. Microparticle suspensions were placed under orbital rotation for 1.5 h (HulaMixer, Invitrogen, Thermo Fisher Scientific, Waltham, MA, USA) during the radiolabeling process.

The inclusion-labeled CaCO_3_ microparticles were prepared by rapidly mixing equal volumes of 0.33 or 1 M CaCl_2_ solution containing ^224^Ra at the target radioactivity level and 0.004–0.3 w/w% Ba^2+^ (relative to CaCO_3_) with 0.33 or 1 M Na_2_CO_3_ solution containing 0–0.7 w/w% SO_4_^2-^ (relative to CaCO_3_) with magnetic stirring or vortexing for 1–3 min. Also for inclusion-labeled microparticles, surface coating was applied in some experiments by addition of PAA towards the end of the crystallization process. The mass amount of the CaCO_3_ microparticles produced was determined by assuming the quantitative yield of the precipitation process.

For both radiolabeling procedures, excess radiolabeling solution was removed prior to the CaCO_3_ microparticles being washed twice with 0.9% saline, sucrose solution or water to remove any ^224^Ra not bound to the particles.

Non-radioactive CaCO_3_ microparticles were also prepared for some experiments through a mock labeling process following the same protocol as for surface-labeling but without the addition of ^224^Ra.

### Radioactivity measurements

Gamma-ray spectroscopy was performed using a Hidex Automatic Gamma Counter (Hidex, Turku, Finland) equipped with a 3-inch diameter NaI crystal. The detector was shielded from background radiation with a lead shield a minimum of 55 mm thick (80 mm on the conveyor side). The counts per minute (CPM) was registered to the 60–110 or 65–345 keV detection window. As can be seen in Table 1, the most abundant x and gamma radiation in these energy ranges originate from ^212^Pb. For the analyses of the radioactive samples, it was therefore assumed that the CPM in these detection windows originated only from the ^212^Pb as the contribution from the other nuclides in the series was considered minimal. The activity of the ^212^Pb was determined directly from the CPM in the 60–110 keV window [23], whereas the ^224^Ra activity was determined indirectly based on counts in the 65–345 keV window when the transient equilibrium between the ^224^Ra and ^212^Pb had been established. Transient equilibrium can be assumed > 2 days after the initial ^212^Pb measurement when the sample vial is left sealed. The data used for the ^220^Rn activity determination were acquired with sources at secular equilibrium (> 6 h after separation and > 1 day from radiolabeling/transfer to a new container) so that decay correction to a common reference time was achieved using the half-life of ^212^Pb.

The limit of quantification (LOQ) was set to equal the average CPM plus 10 times the standard deviation of the measurements of a series of blank samples. When the measured CPM for a sample was below the LOQ value, the CPM was set as equal to the LOQ to produce a theoretical maximum value.

### Release of ^220^Rn from open ^224^Ra sources

**Fig 2:**
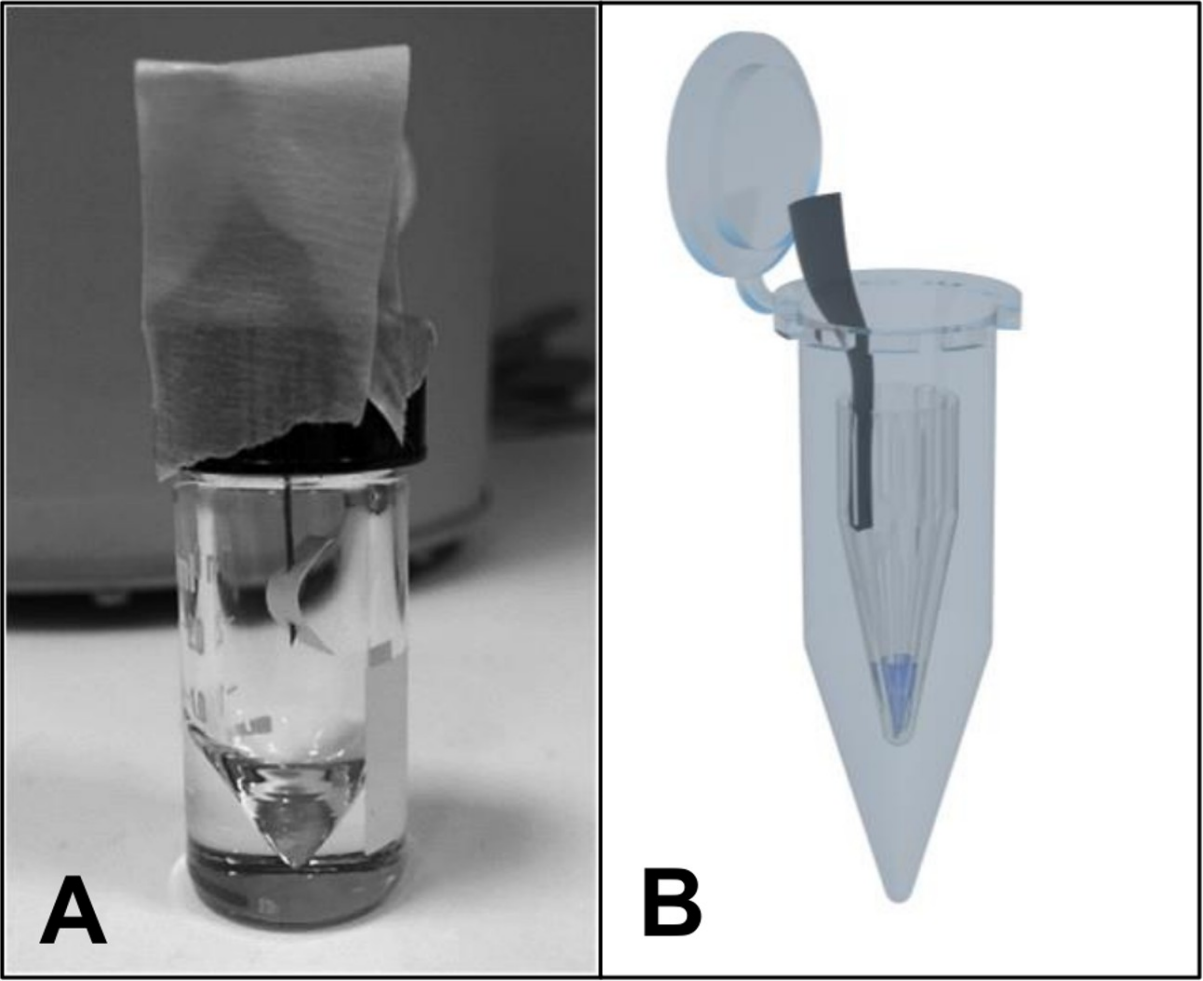
Experimental setups to investigate ^220^Rn diffusion from open sources to air. A 3 mL glass micro reaction vessel (A) and a sealed 5 mL Eppendorf tube (shown open for illustrative purposes) containing a capless 1.5 mL Eppendorf tube (B) were used in separate experiments.

The release of ^220^Rn from open ^224^Ra sources was evaluated with the two different experimental setups as visualized in Fig 2.

The first setup (Fig 2A) aimed at investigating the release of ^220^Rn through the air from a ^224^Ra source. Two μL ^224^RaCl_2_ or 25 μg ^224^Ra-CaCO_3_ microparticles in 2 μL of water suspension were applied on the surface of a small paper strip (1 × 1.5 cm, absorbent bench paper) attached to a syringe needle that had previously been inserted through the silicone septum of a 3 mL glass v-vial (Supelco Analytical, Merck) screw cap. A low sample volume was used for the liquid to be immediately absorbed by the paper and evaporate. In this way, potential release of ^224^Ra from the microparticles to the surrounding liquid could be disregarded. Subsequently, the screw cap with the radioactive sample was carefully inserted into the v-vial while avoiding contact between the paper strip and the interior surfaces of the v-vial before the cap was tightened. After approximately 24 h, the paper strip and needle were placed into two separate vials (sample P and N). The cap was put back onto the empty original vial (V) and the radioactivity in the now three vials was measured (time = t_1_). The total ^224^Ra activity applied on the paper strip (A_Ra_) at time of assembly (t_0_) was assumed to equal the decay corrected sum of the activities in samples P, N and V:

$$A_{\mathrm{Ra},t_{1}}=\frac{{\mathrm{CPM}(V+P+N)}_{t_{1}}}{{\mathrm{EF}}_{\mathrm{Ra}}}=A_{\mathrm{Ra},t_{0}}\times e^{‐\lambda_{\mathrm{Ra}}\times \Delta t_{1}}$$

where EF_Ra_ is the efficiency factor (CPM/Bq) for the 65–345 keV window, λ = ln 2/t_½_ and Δt_1_ = t_1_−t_0_. The amount of ^220^Rn release into air was estimated by the measured ^212^Pb activity in the empty original vial (V) divided by the theoretical maximum ^212^Pb activity generated through ^220^Rn decay (A_Pb_) at the time of measurement (t_1_):

$$\%{}^{220}\mathrm{Rn}\;\mathrm{release}=\frac{{\mathrm{CPM}(V)}_{t_{1}}/{\mathrm{EF}}_{\mathrm{Pb}}}{A_{\mathrm{Pb},t_{1}}}\times 100$$

where EF_Pb_ is the efficiency factor (CPM/Bq) for the 60–110 keV window [23] and $A_{\mathrm{Pb},t_{1}}$ was calculated using the Bateman equation:

$$A_{{\mathrm{Pb},t}_{1}}=A_{\mathrm{Pb},t_{0}}\times e^{-\lambda_{\mathrm{Pb}}\times \Delta t_{1}}\times A_{\mathrm{Ra},t_{0}}\frac{\lambda_{\mathrm{Pb}}}{\lambda_{\mathrm{Pb}}-\lambda_{\mathrm{Ra}}}(e^{-\lambda_{\mathrm{Ra}}\times \Delta t_{1}}-e^{-\lambda_{\mathrm{Pb}}\times \Delta t_{1}})$$

with $A_{\mathrm{Pb},t_{0}}=0$. Although ^212^Pb was present in the samples applied to the paper strip, none of this had the ability to translocalize from the paper strip to the inner surfaces of the vial, and it can therefore be disregarded in the calculations above. Two additional measurements on subsequent days were performed to ensure that there was no ^224^Ra contamination in the original vial (V).

The second experimental setup (Fig 2B) sought to investigate the release of ^220^Rn from solutions containing ^224^Ra. Distinct volumes from 5 to 1000 μL of either free cationic ^224^Ra^2+^ in solution (diluted in 0.9% NaCl or water) or a suspension of 4.3 mg surface-labeled PAA-coated CaCO_3_ microparticles in water were added to 1.5 mL Eppendorf tubes with the lids removed. Each sample tube (S) was inserted into a 5 mL Eppendorf tube (O1) and the lid closed. After 1 day, the outer tube was opened, the inner sample tube transferred to a new 5 mL Eppendorf tube (O2) and the radioactivity in both tubes was measured. The amount of ^220^Rn release from the liquid into the air was estimated by the measured ^212^Pb activity in the empty outer tube (O1) divided by the total activity:

$$\%{}^{212}\mathrm{Pb}\;\mathrm{activity}\;\mathrm{detected}=\frac{\mathrm{CPM}(O1)}{\mathrm{CPM}(O1)+\mathrm{CPM}(S\;\mathrm{in}\;O2)}\times 100$$

The procedure was repeated after 3 and 7 days. The trapping efficiency of the ^220^Rn in the Eppendorf tubes was verified in a separate experiment and found to be more than 99.8%. In this experiment, a sample containing approximately 50 kBq ^224^Ra in a 1.5 mL Eppendorf tube was contained in a sealed zip lock plastic bag for 1 or 7 days before the radioactivity in the plastic bag was measured without the Eppendorf tube inside. Potential release of ^224^Ra to the solution was not taken into account because previous experiments showed that the retention of ^224^Ra on surface-labeled CaCO_3_ microparticles was above 97% *in vitro* [21].

### Adsorption of ^212^Pb onto CaCO_3_ microparticles

To investigate the chemical fate of ^212^Pb subsequent to its release caused by ^220^Rn gas diffusion, a set of experiments was conducted to examine whether the ^212^Pb could be re-adsorbed onto the CaCO_3_ microparticles.

In a pilot experiment, duplicate samples of 5 mg surface-labeled CaCO_3_ microparticles in 0.4 mL sucrose solution were added to a dialysis device (Slide-A-Lyzer MINI Dialysis Device, 0.5 mL format, 20 kDa MWCO, Thermo Fisher Scientific). The device was placed into a conical 15 mL centrifuge tube pre-filled with a suspension of 50 mg non-radioactive CaCO_3_ microparticles in 14 mL Dulbecco’s PBS (pH 7, Gibco, Fisher Scientific), and the tube was then capped with a screw lid. The tube was gently shaken at 150 rpm using a table orbital shaker for 24 h at room temperature before the dialysis device was removed and the tube centrifuged to collect the microparticles in the external solution. The radioactivity levels in the dialysis device (A_D_), the supernatant of the external solution (A_S_) and the pelleted microparticles from the 15 mL tube (A_P_) were measured. The percentage of released ^212^Pb during the 24 h was estimated as follows:

$$\%{}^{212}\mathrm{Pb}\;\mathrm{released}=\frac{\mathrm{CPM}(A_{P}+A_{S})}{\mathrm{CPM}(A_{D}+A_{S}+A_{P})}\times 100$$

The percentage of the released ^212^Pb activity that was adsorbed onto the CaCO_3_ particles was estimated as:

$$\%{}^{212}\mathrm{Pb}\;\mathrm{adsorbed}=\frac{\mathrm{CPM}(A_{P})}{\mathrm{CPM}(A_{P}+A_{S})}\times 100$$

The dependency of the adsorption of ^212^Pb on the CaCO_3_ microparticle concentration was examined in further experiments with a more simplified setup. In this case, ^212^Pb was used directly and not as in the previous experiments where the source of ^212^Pb was the great-grandparent nuclide ^224^Ra. Samples of non-radioactive CaCO_3_ microparticles in 75% Dulbecco’s PBS and 25% fetal bovine serum solution (pH 7.5–8.5) with concentrations ranging from 0.1–50 mg/mL were prepared in 1.5 or 5 mL Eppendorf tubes. Equal activities of ^212^Pb were added to each sample before stirring with orbital motion at 450 rpm using an Eppendorf C thermomixer or at 30 rpm using a HulaMixer at 37°C. After 45–95 min, the samples were centrifuged to separate the microparticles from the solution. The radioactivity levels in the supernatant (A_S_) and the pelleted microparticles (A_P_) were measured, and the percentage of ^212^Pb activity that had adsorbed onto the originally non-radioactive CaCO_3_ microparticles was determined as described above.

The ^212^Pb was produced and separated from the ^224^Ra via ^220^Rn emanation [24] using a single chamber diffusion system [23]. A few μL of ^224^RaCl_2_ solution were distributed on quartz wool (ProQuarz, Mainz, Germany) that was fixed on the inside of the screw cap of a 100 mL glass flask (Simax-Kavalierglass, Prague, Czech Republic). The sealed flask was left inverted overnight in a fume hood for the ^220^Rn to be released through the air inside the vial. The ^220^Rn would then decay into ^212^Pb and become deposited on the interior walls of the container. After 20 to 28 h, the cap with the ^224^Ra source was carefully removed, avoiding the ^224^Ra contamination of the vial. The ^212^Pb was subsequently retrieved by washing the glass walls with 1 M HCl solution.

### Therapeutic effect of surface- and inclusion-labeled ^224^Ra-CaCO_3_ microparticles in mice

Female athymic nude mice (Hsd:Athymic Nude-*Foxn1*^*nu*^, bred at the Department of Comparative Medicine, The Norwegian Radium Hospital, Oslo University Hospital, Oslo, Norway) of 4–6 weeks of age at the start of the experiment were used. The animals were maintained under pathogen-free conditions with food and water supplied *ad libitum* and monitored for changes in body weight, behavior, posture and appearance throughout the study. All procedures involving animals were approved by the Norwegian Food Safety Authority (permit ID 7274) and performed in compliance with regulations set by the same authority and EU Directive 2010/63/EU on the protection of animals used for scientific purposes.

Human ovarian epithelial carcinoma cell line ES-2 (American Type Culture Collection, Wesel, Germany) was cultured in McCoy’s 5A medium (Gibco, Fisher Scientific) supplemented with 10% fetal bovine serum (Gibco, Fisher Scientific) and 1% penicillin/streptomycin (Gibco, Fisher Scientific) at 37°C in a humid atmosphere with 5% CO_2._ The cells were harvested with TrypLE Express solution (Gibco, Fisher Scientific), suspended in cold RPMI 1640 growth medium (Gibco, Fisher Scientific) and kept on ice until inoculation.

The therapeutic effects of four different variants of ^224^Ra-CaCO_3_ microparticles were investigated: both surface- and inclusion-labeled microparticles each with and without PAA coating. A total of 40 mice were randomized to the experimental groups and inoculated IP with 1 × 10^6^ ES-2 cells. One day later, the mice were given the different treatments as shown in Table 2. All the animals that were treated with ^224^Ra-CaCO_3_ microparticles received a single IP injection of 0.29–0.52 mL to achieve the same radioactivity dose based on their body weight. The control animals received 0.9% NaCl (0.4 mL) or 5 mg CaCO_3_ microparticles (0.4 mL) dispersed in sucrose solution.

**Table 2:** Overview of the experimental groups included in the study investigating the therapeutic efficacy of ^224^Ra-CaCO_3_ microparticles in mice.

Experimental group	^224^Ra-labeling method	PAA coating	Activity dose (kBq/kg bodyweight)	CaCO_3_ mass dose (mg per mouse)	No. of mice
0.9% NaCl	n/a	n/a	n/a	n/a	7
CaCO_3_ microparticles	n/a	Yes	n/a	5.0	4
^224^Ra-CaCO_3_ microparticles	Surface	No	350	4.6 ± 0.5	5
^224^Ra-CaCO_3_ microparticles	Surface	Yes	138	4.3 ± 0.4	8
^224^Ra-CaCO_3_ microparticles	Inclusion	No	179	5.5 ± 0.5	8
^224^Ra-CaCO_3_ microparticles	Inclusion	Yes	474	5.6 ± 0.5	8

PAA: polyacrylic acid, n/a: not applicable.

Therapeutic effect was evaluated by the time it took to reach the pre-determined humane endpoints, which were defined as rapid body weight loss (> 10% within one week), ascites build-up that severely impaired mobility and/or cachexia. Mice were euthanized by cervical dislocation when they reached the predetermined endpoint and necropsied for gross pathological examination.

### Statistical analysis

All statistical analyses were performed in GraphPad Prism (version 8.2.1, GraphPad Software, La Jolla, CA, USA) using a significance level of 0.05. The release of ^220^Rn from different ^224^Ra sources was analyzed by Kruskal-Wallis test using the Dunn method to correct for multiple comparisons. Survival curves were compared pairwise by log-rank tests and the Holm-Sidak method to adjust the p-values for multiple comparisons.

## Results

### Release of ^220^Rn to air from open ^224^Ra sources

**Fig 3:**
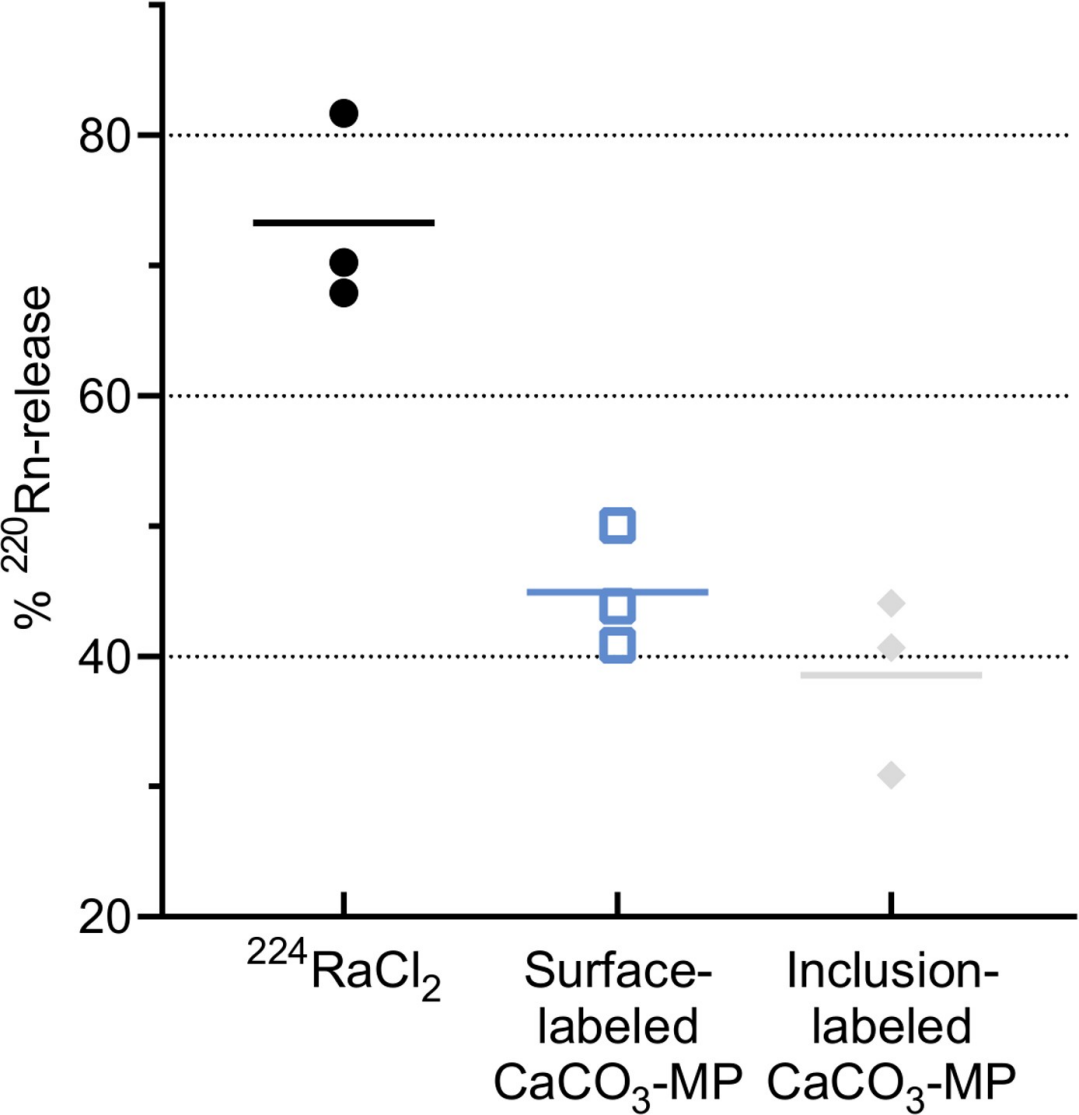
Release of ^220^Rn to air from various open ^224^Ra microsources. The ^220^Rn release was estimated indirectly from measurements of the ^212^Pb activity that had re-localized due to ^220^Rn diffusion. Each independent sample is indicated with a symbol, and a horizontal line represents the average of these three.

The release of ^220^Rn to air from open ^224^Ra microsources was measured indirectly through the amount of daughter ^212^Pb that had re-localized. Radium-224, either in the form of free cation or as surface- or inclusion-labeled CaCO_3_ microparticles, was applied on a paper strip fixed on a needle suspended in a glass vial (Fig 2A). The percentage of ^220^Rn release was estimated by dividing the ^212^Pb activity detected in the outer vial with the theoretical maximum amount of ^212^Pb generated from ^220^Rn decay. The results displayed in Fig 3 show higher ^220^Rn release from ^224^Ra as a free cation than as ^224^Ra-labeled CaCO_3_ microparticles, although the difference was not significant (Kruskal-Wallis, p ≥ 0.0512). No evident difference was seen between the different ^224^Ra-labeling methods of the CaCO_3_ microparticles (p ≥ 0.9999).

**Fig 4:**
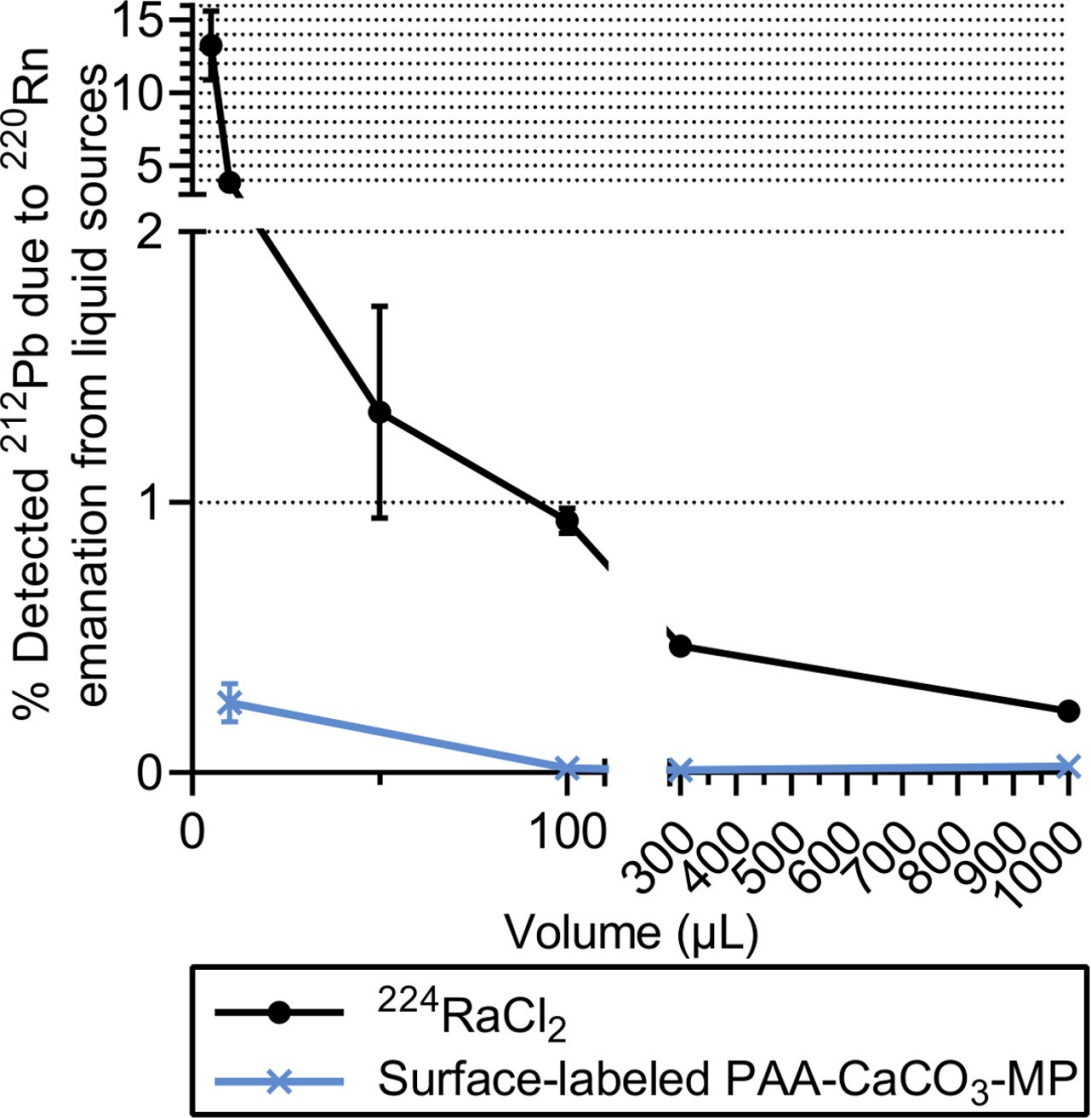
Detected ^212^Pb due to ^220^Rn release from open liquid sources of ^224^Ra approximately 1 day after assembly. Sample volumes ranged from 5 to 1000 μL of either free cationic ^224^Ra or suspensions with 4.3 mg PAA-coated CaCO_3_ microparticles surface labeled with ^224^Ra. Error bars represent standard deviation.

The emanation of ^220^Rn was also evaluated for open liquid sources of ^224^Ra (Fig 2B). Different volumes of free ^224^Ra or surface-labeled PAA-coated CaCO_3_ microparticles were added to a tube without a cap that was contained inside a larger closed tube. After approximately 1 day, the ratio of ^212^Pb activity detected in the outer vial to the total ^212^Pb activity was used to indicate the ^220^Rn release from the open liquid sources. The results show that the ^220^Rn release was at least 4 times lower when the ^224^Ra was adsorbed onto the microparticles as compared with as a dissolved cation, which is in line with the findings from the first experimental setup. The re-localization of ^212^Pb due to ^220^Rn diffusion also appears to be dependent on the liquid volume of the sample, with higher ^220^Rn release at lower volumes (Fig 4). The release at low volumes may be underestimated because of the ^212^Pb activity deposited on the inner tube wall (Fig 2B). The experiment was repeated on days 3 and 7, yielding similar results for the volume dependency (S1 Fig), which indicates that a steady state was obtained after 1 day.

### Adsorption of the ^220^Rn daughter ^212^Pb on CaCO_3_ microparticles

In order to investigate whether the ^212^Pb released from the surface-labeled ^224^Ra-CaCO_3_ microparticles could re-adsorb onto the microparticles, both the percentage of the ^212^Pb activity released from the dialysis unit to the outer solution and the percentage of the ^212^Pb adsorbed onto the originally non-radioactive microparticles were measured. Of the approximately 6% ^212^Pb that had crossed the dialysis barrier, 75% was found to have re-associated with the CaCO_3_ microparticles.

**Fig 5:**
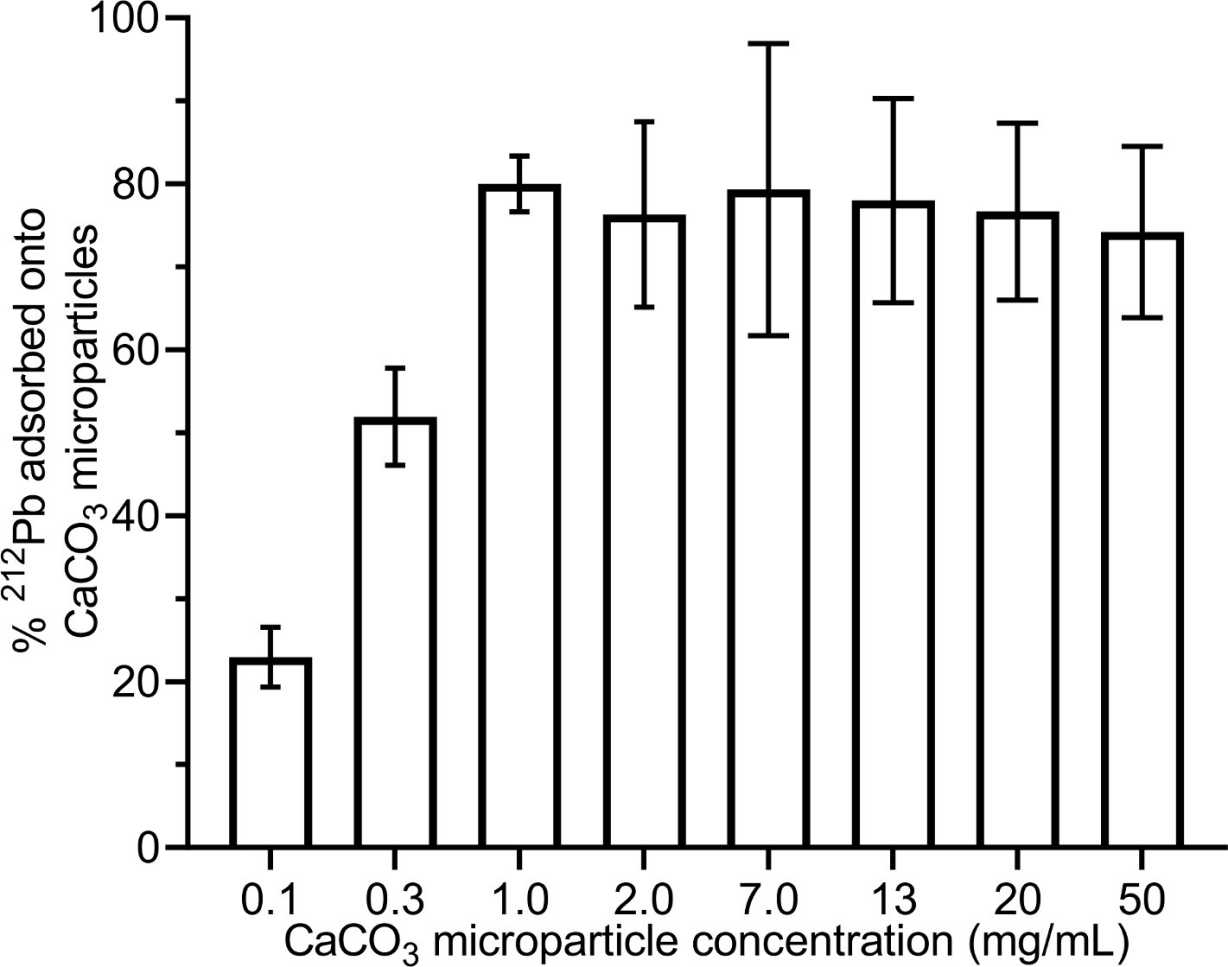
The percentage of ^212^Pb activity adsorbed onto CaCO_3_ microparticles at different CaCO_3_ microparticle concentrations.

Subsequent experiments showed that the degree of ^212^Pb adsorption was high even at relatively low CaCO_3_ microparticle concentrations (Fig 5). Adsorption decreased at CaCO_3_ microparticle concentrations below 1 mg/mL, whereas between 1 and 50 mg/mL, it appeared to reach a plateau with adsorption of approximately 70–80%.

### Therapeutic effect of surface- and inclusion-labeled ^224^Ra-CaCO_3_ microparticles in mice

**Fig 6:**
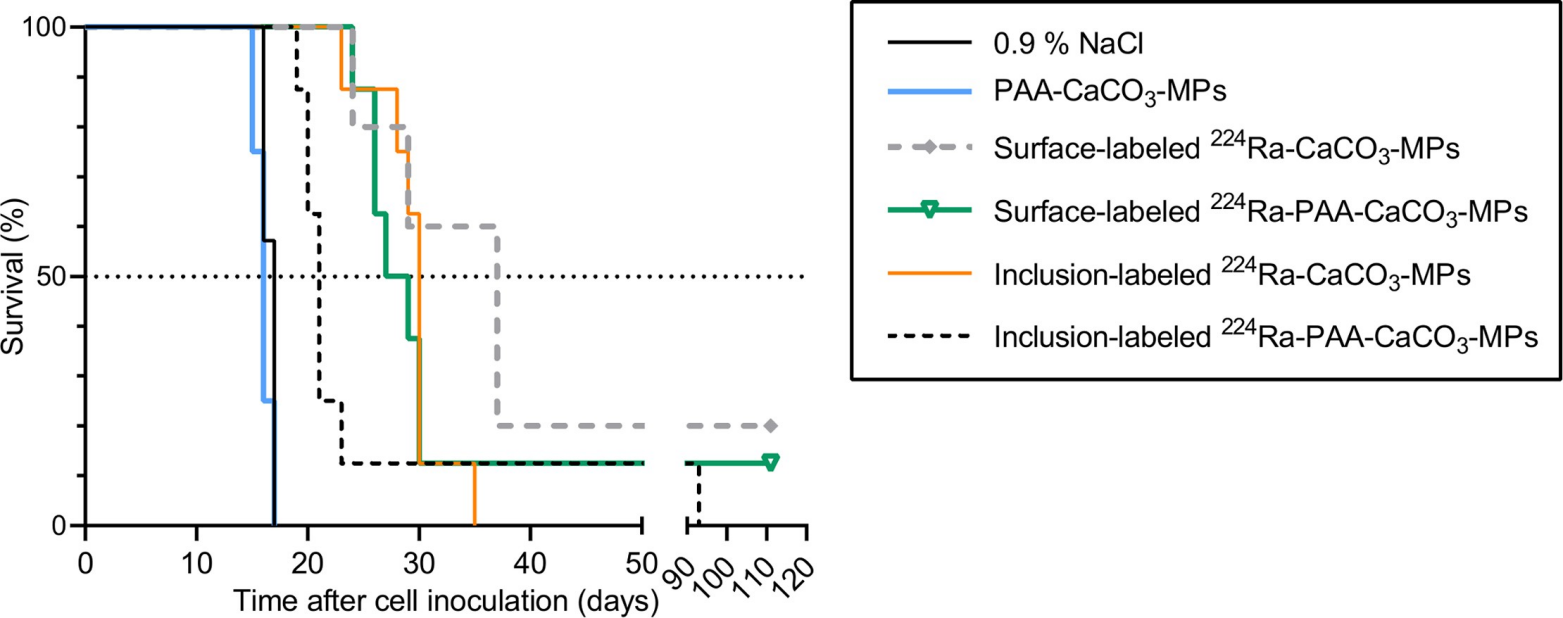
Kaplan-Meier survival curves of athymic nude mice inoculated intraperitoneally with 1 × 10^6^ ES-2 cells and treated 1 day later with intraperitoneal injections of 0.9% NaCl, PAA-coated CaCO_3_ microparticles or ^224^Ra-CaCO_3_ microparticles both with and without PAA coating with an activity dose ranging from 138–474 kBq/kg body weight. N = 4–8 animals per group.

A single IP injection of ^224^Ra-CaCO_3_ microparticles significantly improved survival as compared with both the saline and non-radioactive CaCO_3_ microparticle groups (p ≤ 0.023), regardless of the different radiolabeling methods and PAA coating (Fig 6). The control groups had no survivors beyond day 17, whereas all mice were alive at this time in the different ^224^Ra-CaCO_3_ microparticle groups. No statistically significant difference was found between the surface- and inclusion-labeled products (p ≥ 0.1868), although the survival curves indicate that treatment with the inclusion-labeled ^224^Ra-CaCO_3_ microparticles with a PAA coating had a slightly inferior effect as compared with the other ^224^Ra-labeled microparticle treatments. The survival curves of the saline control group and the group receiving PAA-CaCO_3_ microparticles overlap, showing that the microparticle carrier itself had no effect in this cancer model.

## Discussion

The current study demonstrates that there is a significant diffusion of ^220^Rn from ^224^Ra-CaCO_3_ microparticles. Radon emanation from mineral grains is assumed to be governed by alpha recoil because diffusion through the solid matrix can be considered negligible [25, 26]. When ^220^Rn is generated by alpha decay of ^224^Ra (Fig 1), the atom acquires a kinetic energy of approximately 100 keV [27] resulting in a recoil range below 50 nm in most solids [28]. Hence, ^220^Rn can only escape from the CaCO_3_ microparticles if the ^224^Ra atom upon decay is located closer than the recoil distance to either the outer surface of the microparticle or the surface of an internal pore connected to the outer surface, such that radon can subsequently diffuse through the pore volume and out from the microparticle.

The degree of ^220^Rn diffusion seemed to be relatively independent of the radiolabeling method, that is, whether ^224^Ra was adsorbed onto the surfaces or incorporated into the bulk of the microparticles during CaCO_3_ precipitation. Based on the established theory for radon emanation from mineral grains, this may be explained by a porous structure of these CaCO_3_ microparticles that allows ^220^Rn to escape. SEM images presented in S1 Table indicate a degree of porosity of CaCO_3_ microparticles, both with and without PAA coating. This is also in line with literature, where CaCO_3_ microparticles synthesized by a similar procedure were shown to be highly porous, as approximately 40% of the volume of the microparticles was estimated to be internal pores [29]. The comparable ^220^Rn release from the differently ^224^Ra-labeled CaCO_3_ microparticles is further corroborated by a relatively high radon diffusion coefficient in limestone [30], a mineral mostly composed of various crystal forms of CaCO_3_. The average distance ^220^Rn can travel is dependent upon half-life (55.8 s, Fig 1) and the diffusion coefficient of the material the radon atoms traverse. Typically, the mean diffusion range is estimated to be a few hundred micrometers in water and centimeters in air (Table 3). The estimated mean distance of 5.2 mm that ^220^Rn can travel in limestone is thus approximately 1000 times greater than the median diameter of the CaCO_3_ microparticles examined (range from 3 to 7 μm, S1 Table) and indicate low attenuation of radon diffusion within the microparticles.

**Table 3:** Overview of radon diffusion coefficients and resulting mean diffusion lengths in different materials.

Material	Temperature (°C)	Diffusion coefficient (cm^2^/s)	Mean ^220^Rn diffusion length* (mm)
Water	37	1.9 × 10^−5^ [20, 31]	0.4
Water	18	1.1 × 10^−5^ [32, 33]	0.3
Water	n/a	1 × 10 ^−5^ [28]	0.3
Air	n/a	0.1 [28]	28
Limestone (CaCO_3_)	n/a	3.4 × 10^−3^ [30]	5.2

* The mean diffusion length is given by: $\sqrt{\frac{D}{\lambda }}$, where D is the diffusion coefficient and λ = $\frac{\ln(2)}{t_{1/2}}$ is the decay constant of ^220^Rn.

The high diffusion coefficient of radon in limestone also implies that diffusion rates should be similar from ^224^Ra-CaCO_3_ microparticles and cationic ^224^Ra. However, both the air and liquid phase studies demonstrated that the diffusion of ^220^Rn was reduced when ^224^Ra was bound to microparticles as compared with free ^224^Ra. One explanation for this difference may be that release of ^220^Rn through alpha recoil into a pore space also can lead to embedding of the radon atom into an adjacent grain [26]. If the residual kinetic energy of the recoiling radon atom is sufficient to traverse the internal pore diameter of the microparticles, the result can be a re-trapping of ^220^Rn in the solid microparticle matrix. The probability of implantation of recoiling radon atoms will be higher if pores are filled with air compared to water and is also dependent on the pore size [34].

All variants of the ^224^Ra-CaCO_3_ microparticles significantly extended the survival of the mice with IP tumors, but a correlation between the effect and the parameters that were varied was not clear. One treatment, inclusion-labeled ^224^Ra-CaCO_3_ microparticles with a PAA coating, seemed to be slightly less effective. Because of some variations in the ^224^Ra-labeling yield, the activity dose was not directly comparable in the four different ^224^Ra-CaCO_3_ microparticle groups. The highest activity dose (474 kBq/kg) was administered to the mice receiving inclusion-labeled ^224^Ra-CaCO_3_ microparticles with a PAA coating. Therefore, this does not explain why this variant of the ^224^Ra-CaCO_3_ microparticles appeared less effective and may instead indicate a potential reduction of ^220^Rn diffusion from the microparticles caused by the polymer surface coating. The surface-labeled PAA-coated variant would on the other hand not be affected by this, because surface labeling was performed after the microparticles were coated with the polymer and not prior to, as was the case for the inclusion-labeled. The surface-labeled ^224^Ra-CaCO_3_ microparticles were given at an activity dose approximately twice as high (350 kBq/kg) as the analog with the PAA coating (138 kBq/kg) and the inclusion-labeled without (179 kBq/kg). Previous studies in the same tumor model showed prolonged survival with increasing administered activity [11, 15]; however, the difference was not statistically significant between activity doses of 150 and kBq/kg [11]. Free ^224^Ra was not used as a control in mice due to rapid translocalization from the peritoneal cavity [21] which resulted in inferior therapeutic efficacy compared to ^224^Ra-CaCO_3_ microparticles, even at 25% higher radioactivity dose [15].

**Fig 7:**
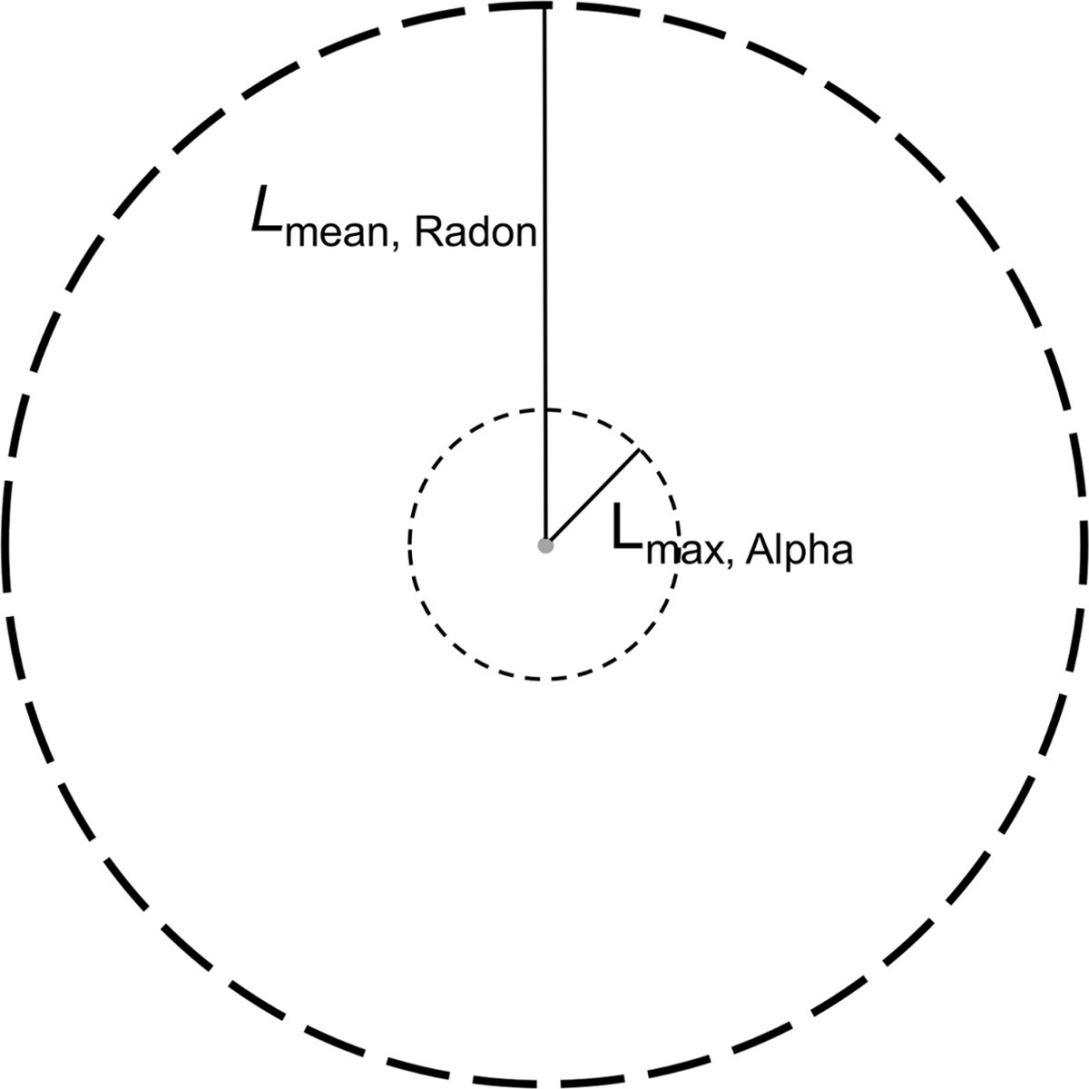
Illustration of the maximum distance alpha particles can travel in water (L_max, Alpha_ = 100 μm) and estimated mean diffusion length of ^220^Rn in water (L_mean, Radon_ = 300–400 μm) relative to the CaCO_3_ microparticle size (5 μm in diameter).

The release of ^220^Rn from CaCO_3_ microparticles affects the microdistribution of the alpha particles from the ^224^Ra series. The distance ^220^Rn can travel subsequent to its escape from the microparticles can be estimated by its mean diffusion length (Table 3). In the case of using the ^224^Ra-CaCO_3_ microparticles as a treatment for IP cancer when the intent is to irradiate liquid volumes and serosal surfaces in the peritoneal cavity harboring micrometastases, the diffusion distance in water is probably the most relevant to consider. The additional distance ^220^Rn atoms can travel in water because of recoil energy is estimated to be only 0.09 μm [28], which is significantly shorter than the 300–400 μm ^220^Rn on average diffuses in the same material and was therefore disregarded. As illustrated in Fig 7, ^220^Rn diffusion can result in an increase in the irradiated volume from ^224^Ra-CaCO_3_ microparticles. The maximum distance an alpha particle can travel in water is less than one third of the mean diffusion length of ^220^Rn in the same medium. Thus, the irradiated volume can be increased by a factor of 27 through ^220^Rn diffusion. This indicates that the alpha-particle related microdosimetry of ^224^Ra-labeled microparticles may be significantly different from that of microparticles labeled with single-step decaying alpha-emitting radionuclides.

If only alpha-particle radiation is considered significant for the biological activity of ^224^Ra-CaCO_3_ microparticles, then three out of four alpha particles are produced by ^220^Rn and its progenies. Depending on the degree of radon diffusion from the microparticles, a significant fraction of the therapeutic radiation dose can be delivered beyond the alpha-particle range from a microparticle. Thus, the emanation of ^220^Rn could be of benefit both for extending the effective alpha-particle range and in terms of radiation “dose smoothening” as the microparticles may not be perfectly distributed in the treated cavity. The brachytherapy application DaRT also exploits the daughter nuclides of ^224^Ra for extending the effective alpha-particle range. Through modeling, it has been shown that a point source of ^224^Ra (with approximately 100 kBq) placed in a solid tumor with approximately 40% of the radon being released results in therapeutic alpha-particle dose levels over a distance of 4–7 mm in diameter [20]. This distance is also in line with preclinical studies in which necrotic regions of 5–7 mm in diameter have been observed after the placement of a single ^224^Ra wire into squamous cell carcinoma tumors in mice [13].

The diffusion of ^220^Rn from CaCO_3_ microparticles also raises questions about the fate of the subsequent progenies. The immediate daughter of ^220^Rn, ^216^Po, has a half-life of only 0.15 s and will decay essentially in the same location as the mother nuclide. However, the subsequent progeny, ^212^Pb, has a sufficiently long half-life to allow it to be transported further away from the parent nuclide and even redistribute from the peritoneal cavity. Approximately 30% of the energy released from alpha particles in the ^224^Ra decay chain originate from progenies of ^212^Pb. Hence, a significant fraction of the therapeutic radiation dose can be lost if this radionuclide decays away from the target area. This is the case in the DaRT application, where a considerable fraction of ^212^Pb (assumed to be between 30 and 50% [20, 35]) leaves the tumor via systemic circulation and redistributes to distant organs and tissues. With this in mind, a particularly interesting feature of ^224^Ra-CaCO_3_ microparticles is their ability to adsorb the free-floating ^212^Pb generated following ^220^Rn escape from the microparticles. The data from liquid phase studies indicate that the adsorption of ^212^Pb onto the microparticles occurs to a significant degree, even under conditions mimicking an *in vivo* environment. The adsorption was also high over a wide range of microparticle concentrations, indicating that this phenomenon can also occur in the clinical treatment setting.

An understanding of which factors that impact the therapeutic effect is important when developing a new radiopharmaceutical. For the surface-labeled ^224^Ra-CaCO_3_ microparticles we have previously shown that the antitumor activity was dependent on the administered activity [11, 15]. In addition, the results supported a positive correlation between therapeutic effect and specific activity, defined as the ratio of activity to mass dose of CaCO_3_, and a negative correlation between specific activity and degree of ^224^Ra retention on the microparticles *in vivo* [15], altogether indicating that the therapeutic effect is not solely dependent on the total activity dose. The results presented in the current study suggest that ^220^Rn diffusion from the microparticles and re-adsorption of ^212^Pb may play a role. Further investigations are needed to elucidate the relationship between these different factors.

## Conclusion

The ^220^Rn diffusion from ^224^Ra-labeled CaCO_3_ microparticles is significant yet reduced as compared with the release from cationic ^224^Ra. Furthermore, the diffusion of ^220^Rn from microparticles seem to be independent on whether the microparticles were labeled by the surface adsorption of ^224^Ra or if the ^224^Ra was incorporated into the bulk of the microparticles. There is a significant adsorption of ^212^Pb, the ^220^Rn daughter with the longest half-life, onto CaCO_3_ microparticles even at microparticle concentrations of a few mg/mL. Thus, the release of ^220^Rn and re-adsorption of ^212^Pb are features that may have implications for the radiotherapeutic use of ^224^Ra-labeled CaCO_3_ microparticles. The diffusion of ^220^Rn up to a few hundred micrometers can extend the effective range of the inherent short-range alpha particles and may cause a “dose-smoothening effect” to counteract potential heterogeneous distribution of microparticles in the treated cavity, while the re-adsorption of ^212^Pb onto the CaCO_3_ microparticles can contribute to enhancing the retention of ^212^Pb in the target area.

## Supporting information

S1 File Raw data. (XLSX)

S1 Table Size distribution and SEM images of CaCO_3_ microparticles with and without PAA surface coating. (PDF)

S1 Fig Detected ^212^Pb due to ^220^Rn release from open liquid sources of 224Ra approximately 3 (A) and 7 days (B) after assembly. The sample volumes ranged from 5 to 1000 μL of either free cationic 224Ra or suspensions with 4.3 mg PAA-coated CaCO3 microparticles surface labeled with 224Ra. Error bars represent standard deviation.
(TIF)
