# Supplementary material for: Radon-220 diffusion from 224Ra-labeled calcium carbonate microparticles: Some implications for radiotherapeutic use
Source: PLoS One. 2021 Mar 4;16(3):e0248133. doi: 10.1371/journal.pone.0248133 (PMC7932545; doi:10.1371/journal.pone.0248133)
Supplement: S1 Table — (PDF) [file pone.0248133.s002.pdf]

**S1 Table. Size distribution and SEM images of $CaCO_3$ microparticles with and without PAA surface coating.**

| Description | Volume based diameter* (µm)<br>Dv10 | Dv50 | Dv90 | Observations and SEM image |
|---|---|---|---|---|
| $CaCO_3$ microparticles | 2.0 | 3.8 | 7.2 | 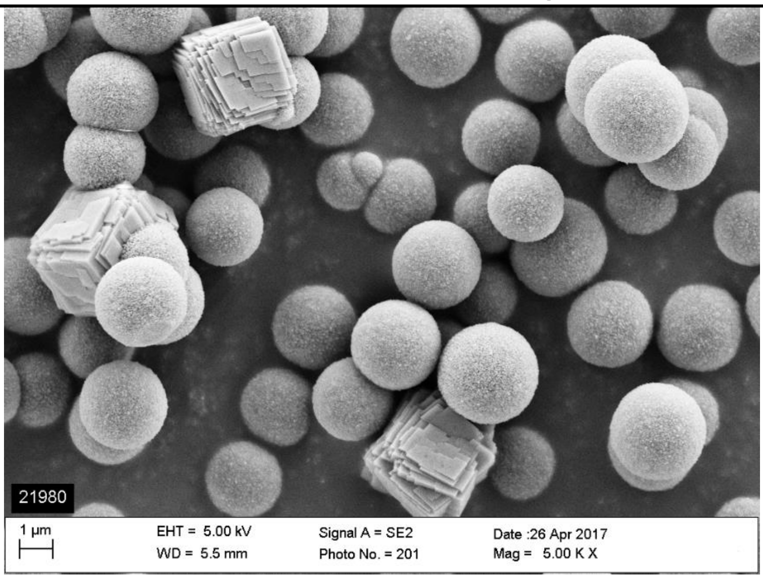<br>Mainly spherical morphology<br>Relatively rough surface structure, but homogenous.<br>Surface structure indicates a degree of porosity |
| $CaCO_3$ microparticles with PAA coating | 1.8 | 3.2 | 5.5 | 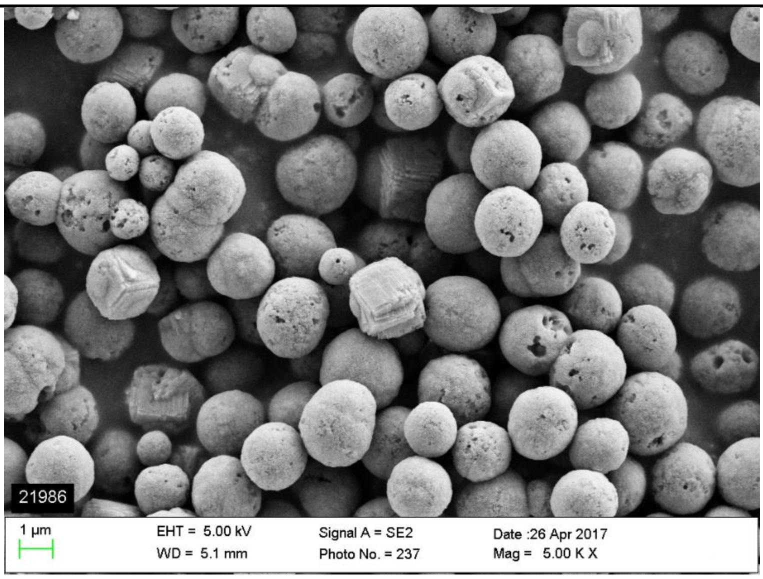<br>Mainly spherical morphology<br>Somewhat smoother surface structure than corresponding $CaCO_3$ microparticles without PAA coating, but more irregular.<br>Surface structure indicates a degree of porosity |

*The size distribution is described by the Dv10, Dv50 and Dv90 values, which represent the volume based diameters where 10, 50, and 90% of the population lie below.
