# Supplementary figures and images for: Radon-220 diffusion from 224Ra-labeled calcium carbonate microparticles: Some implications for radiotherapeutic use
Source: PLoS One. 2021 Mar 4;16(3):e0248133. doi: 10.1371/journal.pone.0248133 (PMC7932545; doi:10.1371/journal.pone.0248133)

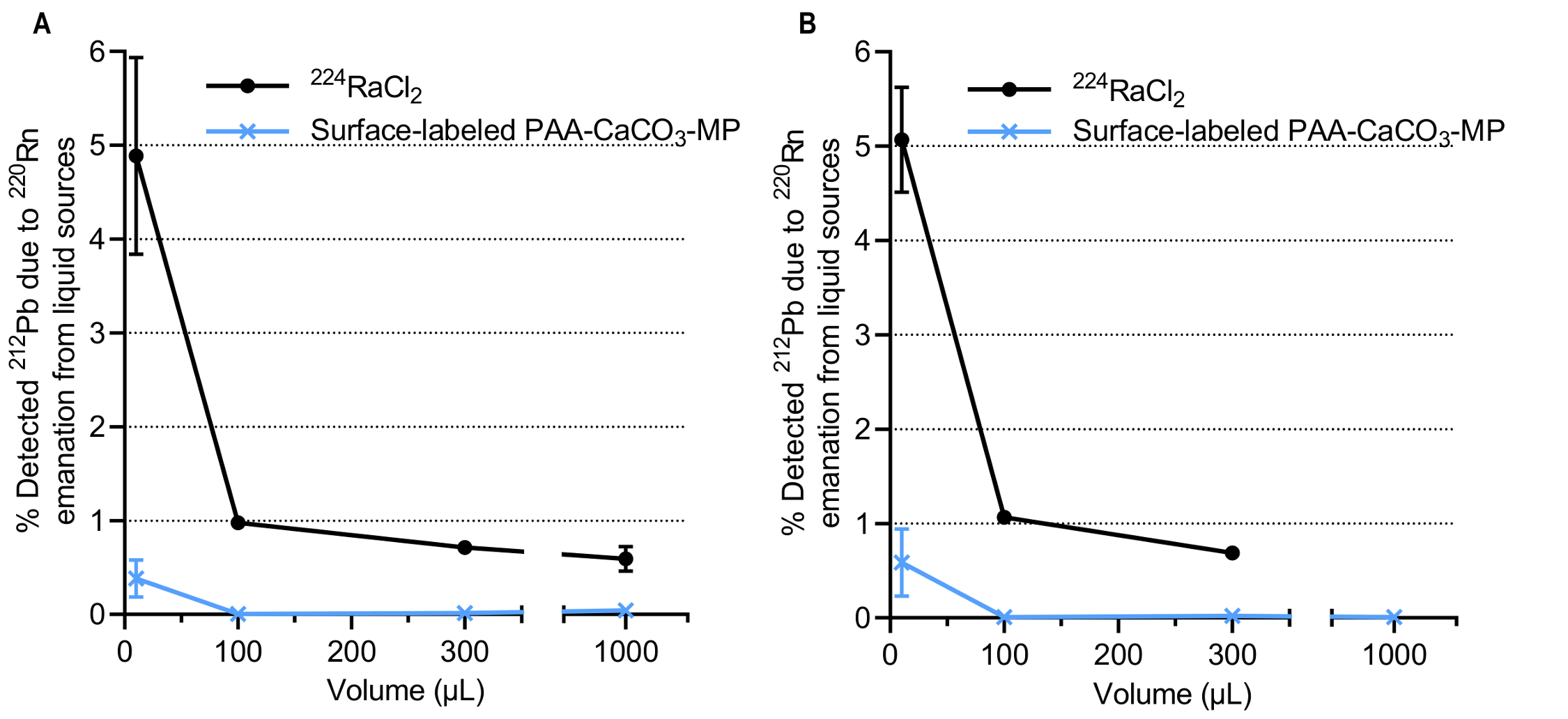

Supplement: S1 Fig — Detected 212Pb due to 220Rn release from open liquid sources of 224Ra approximately 3 (A) and 7 days (B) after assembly. The sample volumes ranged from 5 to 1000 μL of either free cationic 224Ra or suspensions with 4.3 mg PAA-coated CaCO3 microparticles surface labeled with 224Ra. Error bars represent standard deviation. (TIF) [file pone.0248133.s003.tif]
